# The indications for core decompression surgery in patients with ARCO stage I-II osteonecrosis of the femoral head: a new, comprehensive prediction system

**DOI:** 10.1186/s12891-023-06321-0

**Published:** 2023-03-30

**Authors:** Congcong Wei, Meng Yang, Kun Chu, Jia Huo, Xiao Chen, Bo Liu, Huijie Li

**Affiliations:** 1 Department of Joint Surgery, No. 215 Hospital of Shaanxi Nuclear Industry, No.35, West Weiyang Road, Xianyang, Shaanxi Province China; 2 Department of Osteonecrosis and Hip Surgery, the Third Hospital of Hebei Medical University, No.139 Ziqiang Road, Shijiazhuang, Hebei Province P.R. China

**Keywords:** Core decompression, Osteonecrosis, Predictive model, Indication

## Abstract

**Background:**

Core decompression (CD) is considered the most popular treatment method for patients with Association Research Circulation Osseous (ARCO) stage I-II osteonecrosis of the femoral head (ONFH). However, the definitive indication for CD is currently not well established.

**Methods:**

This was a retrospective cohort study. Patients who were diagnosed with ARCO stage I-II ONFH and who underwent CD were included. According to the prognosis, the patients were divided into two groups: collapse of the femoral head after CD and noncollapse of the femoral head. Independent risk factors for the failure of CD treatment were identified. Subsequently, a new scoring system that included all these risk factors was built to help estimate the individual risk of CD failure in patients who were planning to undergo CD.

**Results:**

The study included 1537 hips after decompression surgery. The overall failure rate of CD surgery was 52.44%. Seven independent prognostic factors for failed CD surgery were identified, such as male sex (HR = 75.449; 95% confidence interval (CI), 42.863-132.807), Aetiology (Idiopathic HR = 2.762; 95% CI, 2.016–3.788, Steroid-induced HR = 2.543; 95% CI, 1.852–3.685), if the patient had a seated occupation (HR = 3.937; 95% CI, 2.712–5.716), age (HR = 1.045; 95% CI, 1.032–1.058), haemoglobin level (HR = 0.909; 95% CI, 0.897–0.922), disease duration (HR = 1.217; 95% CI, 1.169–1.267) and the combined necrosis angle (HR = 1.025; 95% CI, 1.022–1.028). The final scoring system included these seven risk factors, and the area under the curve of this scoring system was 0.935 (95% confidential interval = 0.922–0.948).

**Conclusion:**

This new scoring system might provide evidence-based medical proof for determining whether a patient with ARCO stage I - II ONFH might benefit from CD surgery. This scoring system is crucial for making clinical decisions. Consequently, this scoring system is recommended before CD surgery, which could help determine the potential prognosis of patients.

Core decompression (CD) is the most widely used clinical technique for joint preservation, and it significantly reduces the bone marrow pressure, controls pain, and prevents or slows further joint destruction in patients with osteonecrosis of the femoral head (ONFH) [1]. Jie K et al. reported that among other hip-preserving treatment methods, such as pharmacological agents, hyperbaric oxygen, extracorporeal shock wave therapy and proximal femoral osteotomy, CD (and combined with bone grafting) was characterized as having a higher evidence level (Grade B) in the treatment effect of ONFH than other treatments [2]. Consequently, CD is considered the most popular treatment method for patients with ARCO Stage I and II ONFH, which means that the femoral head is in a precollapse status [3].

However, the prognosis of patients, who do not have a hip arthroplasty with artificial joint implantation and who undergo CD, is not always satisfactory [4, 5]. The long-term survival rate of these types of hip joints varies between different cohorts. For instance, D’Ambrosi et al. reported that the survivorship of hips treated with CD was 50% at 75 months of follow-up [6]. The hip survival rate was 80% in patients with Ficat stage I-II ONFH [6]. Serong et al. reported collapse of the femoral head and subsequent treatment failure in 37.2% of patients [7]. Meanwhile, the management of patients with failed CD surgery also represents a great challenge for hip surgeons. Liu et al. reported that individuals with a failed CD surgery had an approximately 3-fold increased risk of periprosthetic femoral fractures [8]. Failure in these cases was attributed to the filling of the decompression tunnel of the femoral neck with sclerotic bone, which finally led to a reduction in the bone strength and increased bone fragility [9]. Finally, this loss of bone strength causes an increased incidence of periprosthetic femoral fractures [10]. Therefore, how to improve the prognosis of patients undergoing CD has become crucial.

Due to the relatively simple surgical technique of CD, the proper selection of patients is the key point to reduce the collapse incidence after CD [11]. In fact, hip surgeons are constantly trying to determine which patients might benefit from CD surgeries. For instance, using the modified method of Kerboul et al., Ha et al. measured the arc of the femoral surface that is necrotic on a midcoronal section as well as on a midsagittal magnetic resonance image and then calculated the sum of the angles. The authors found that the lesion size is associated with an early collapse of the femoral head after CD [12]. Kuroda retrospectively analysed 505 hips from 310 patients diagnosed with ONFH and classified them using the Japanese Investigation Committee (JIC) classification. They demonstrated that the location of the lesion is a certain risk factor that is correlated with CD failure [13]. All these above results indicated that the prognosis of a patient undergoing CD is not affected by a single factor but by multiple factors [14]. But these previous studies have been focused on the overall success or failure rate of a group of patients. However, studies exploring the success or failure rates of individual patient procedures have not. Consequently, the success rate based on a population of CD patients should be well established. However, for one individual, the accurate collapse rate after CD cannot be predicted preoperatively. This is the other limitation of these previous studies.

To fill this gap, we comprehensively collected the radiological data, clinical data and laboratory test results of patients with ARCO stage I-II ONFH who underwent CD. The independent risk factors associated with femoral head collapse after CD were identified, and a diagnostic test model was built to estimate the individual risk. Finally, a scoring system was built to help surgeons determine the potential prognosis of patients undergoing CD, which might provide great assistance for decision making regarding the treatment strategies.

## Patients and method

### Study design

This study employed a retrospective case-control design. Patients who were diagnosed with ARCO stage I-II ONFH and who underwent CD at our hospital from May 2015 to May 2019 were included in our study. We evaluated patients for a period of two years. According to the prognosis, the patients were divided into two groups: patients who had collapse of the femoral head after CD and patients who did not have collapse of the femoral head. The independent risk factors for failure of CD treatment were identified. Subsequently, a new scoring system that included all of these risk factors was built to help estimate the individual risk of CD failure in patients who would undergo CD.

### Participants

The study was approved by the Institutional Review Board of the Third Hospital of Hebei Medical University and was conducted in accordance with the Declaration of Helsinki and the regulations of the Health Insurance Portability and Accountability Act. As this study was retrospective and all patient information was deidentified before the analysis, the requirement for informed consent was waived.

The inclusion criteria were as follows: [1] patients with osteonecrosis of the femoral head aged > 18 and < 70 years; [2] the necrotic hip was classified as stage I or II according to the ARCO system, as determined using preoperative X-ray and magnetic resonance imaging (MRI); and [3] patients who underwent multiple CD operations on one or both hips.

The exclusion criteria were as follows: [1] patients with follow-up for less than two years; [2] fracture of the femoral neck or intertrochanteric fracture during patient follow-up; [3] patients who underwent a surgical intervention to preserve the femoral head before and after CD; and [4] patients without MRI data before CD.

Note that if a patient received bilateral CDs, he or she was considered two independent individuals.

### Data collection

#### Demographic information

The demographic and general information of the patients was identified based on their medical records, and this data included age, sex, BMI, aetiology, side, occupation, smoking and alcohol abuse. In this study, alcohol abuse was defined as the intake of greater than 400 mL of alcohol per week for more than 10 years. Steroid use was defined as a daily dose of greater than 30 mg/kg or a cumulative dose of greater than 2000 mg.

ONFH occurring after alcohol abuse was considered alcohol-induced ONFH. ONFH occurring after steroid use was considered steroid-induced ONFH.

#### Clinical evaluations

Clinical evaluations of the precollapse hips were performed; they were classified based on the classification system of the Association Research Circulation Osseous (ARCO); and the time span between the start of hip symptoms and CD was also determined. The patient’s pain symptoms were assessed before CD based on the visual analogue scale (VAS). The history of patients was also investigated, and factors such as hypertension, Harris hip score, number of channels, diabetes, corticosteroid treatment, osteoporosis, dialysis, vasculitis, arterial thrombosis, immune system diseases, preoperative treatment with hip preservation and preoperative crutch use were evaluated.

#### Laboratory examinations

Blood examinations, including the total cholesterol, triglyceride and haemoglobin levels, measured before the CD were recorded.

#### Radiological measurements

For each patient, anterior-posterior view X-ray examinations of the pelvis and both lower extremities were performed before and after surgery. A computed tomography (CT) scan and magnetic resonance imaging (MRI) of both hips were also performed before surgery. In addition, crescent signs, cystic changes of the femoral head, MRI feature-double-line signs, MRI feature-articular effusion, articular glenoid labral tears and acetabular impingement syndrome were also recorded. The measurement methods of these radiological indicators are listed below.

**Fig. 1:**
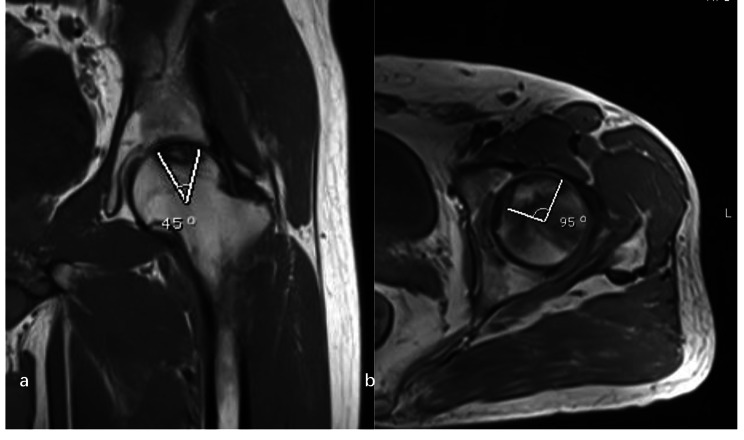
Calculation of the combined necrotic angle from magnetic resonance imaging scans. a: The angle of the necrotic area in the midcoronal image. b: The angle of the necrotic area in the midsagittal image. The combined necrotic angle = a + b

(1) Femoral head radius length. According to a modified method of Aaron et al., circles with the same radius were fitted to the normal portion of the femoral head contour on the serial radiographs of each patient [15]. The femoral head radius was defined as the distance from the centre of the rotation of the femoral head to the border.

(2) MRI feature-intensity. The intensity of the signal on the coronal T1-weighted spin-echo images was classified as Grade ɑ (high), Grade β (mixed), or Grade γ (low) [16].

(3) MRI feature-Sum [16]. Extent: The femoral head profile on the coronal image was seen as an intact circle; The maximum radial distance of the necrotic area from the circumference of the circle was less than one-fourth of the circle diameter was considered to be Grade (A) The distance of one-fourth to one-half of the diameter was classified as Grade (B) The distance of one-half of the diameter or greater was classified as Grade (C) Location: The major weight-bearing rim of the acetabulum was divided into three parts on the coronal image. The measurement of the necrosis area of the articular rim of the adjacent femoral head to less than one-third of the weight-bearing acetabular rim was classified as Grade A. One-third to less than two-thirds was Grade B, and two-thirds or more was Grade C. The extent and location were combined to calculate the MRI-feature SUM. Grade I: Location A/B/C, Extent A or Location A, Extent A/B/C. Grade II: Location B, Extent B/C. Grade III: Location C, Extent B/C.

(4) MRI feature-bone marrow oedema. Marrow oedema was classified as Grade I if its maximum radial distance from the circumference was less than half the diameter of the circle. Marrow oedema was considered to be Grade II if the distance was more than one-half and did not reach the intertrochanteric line. Bone marrow oedema was classified as Grade III if the distance exceeded the intertrochanteric line.

(5) The combined necrosis angle. The necrotic angles was measured on the mid-coronal and mid-sagittal planes [17]. The center of the femoral head and the necrotic angle measured on MRI were digitally identified and measured by a single observer in a picture filing and communication system. The sum of the 2 angles was the combined necrosis angle (Fig. 1).

### Surgical techniques

The surgery for all patients was performed by the same surgical team. All surgeries were performed by the same experienced surgeon. All surgical procedures used for CD were conducted as described by Warner et al. [18]. All procedures were performed under general anesthesia, with skin incision at the tip of the greater trochanter. Before beginning the procedure, the location, size, and boundary of the necrotic area was identified on AP and lateral radiographs. Under the guidance of G-arm fluoroscopy, a guide pin was advanced centrally to the apex of the femoral neck with an appropriate depth and alignment [19]. Next, a cannulated drill bit was used for drilling channel until it reached 5 mm beneath the subchondral bone. Likewise, one or two additional channels were drilled towards the necrotic lesions to reduce intraosseous pressure and stimulate revascularization. No patients were with prophylactic antibiotics preoperatively or postoperatively. Antithrombotic drugs were not administered to patients to prevent blood clots, as activity was used to prevent blood clots. Patients were trained in bed for lower limb flexion, extension and leg raising until one month postoperatively.

Following surgery, patients were allowed to bear 50% of the weight they would have carried on one leg in their previous healthy state on the affected leg for 6 weeks. The patients were unable to squat on the affected leg and must use crutches during this 6 week period. After 6 weeks, patients were allowed to progress to full weight-bearing. Patients were then provided abductor strengthening exercises and educated to avoid high-impact activities for 1 year. Rehabilitation throughout recovery to include hip abductor strengthening and ROM exercises should be performed as early as possible after surgery. If patients were asymptomatic at 10–12 months postoperatively with no radiographic evidence of collapse, they were allowed to resume all usual activities, including higher impact loading activities (such as running).

### Outcome of interest

**Fig. 2:**
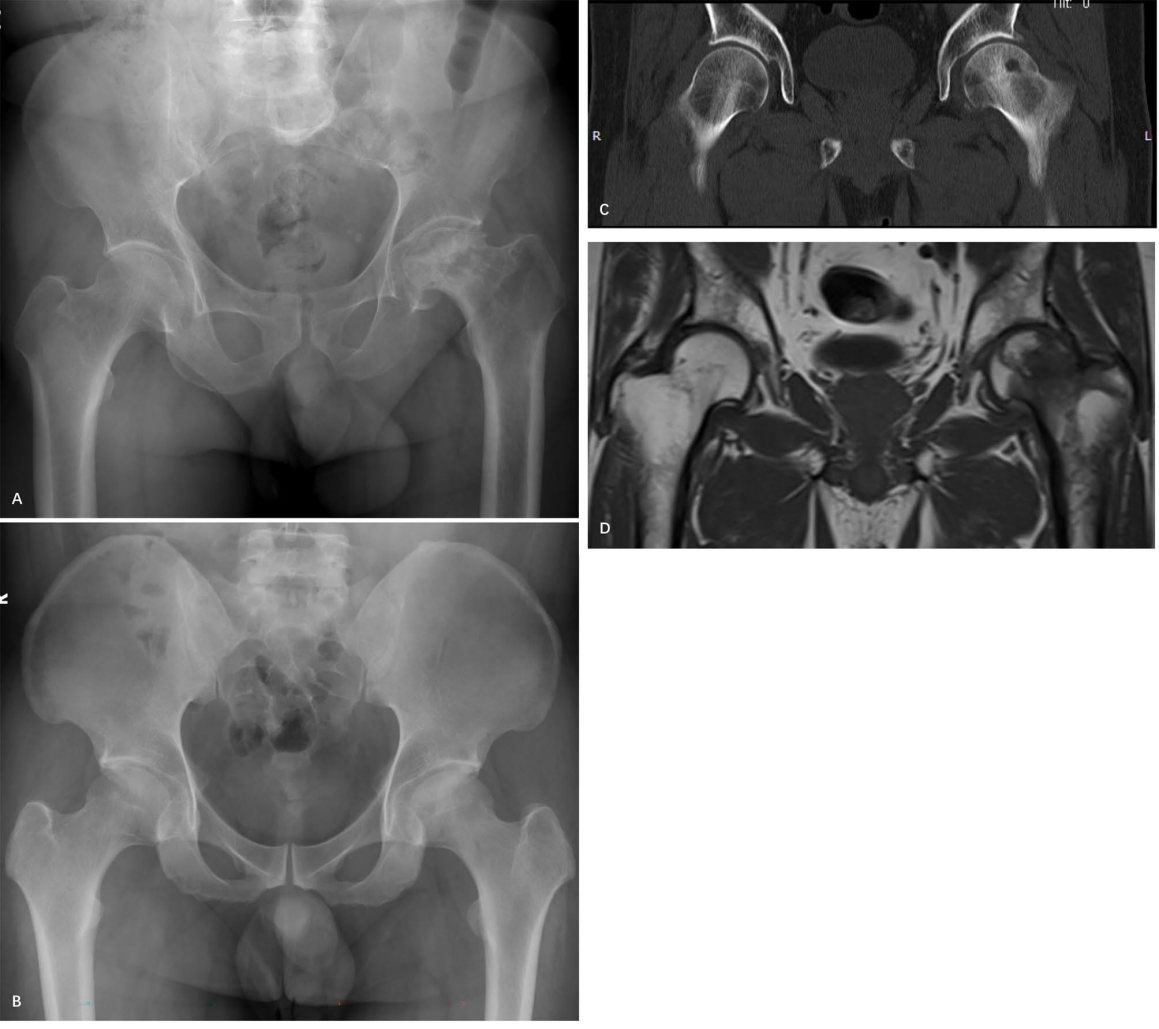
A 60-year-old male patient with an ONFH of his left hip. The patient had undergone CD surgery 2 years previously. (A) The radiograph showed flattening of his left femoral head. (B) Preoperative X-rays. (C) Preoperative CT. (D) Preoperative MRI.

**Fig. 3:**
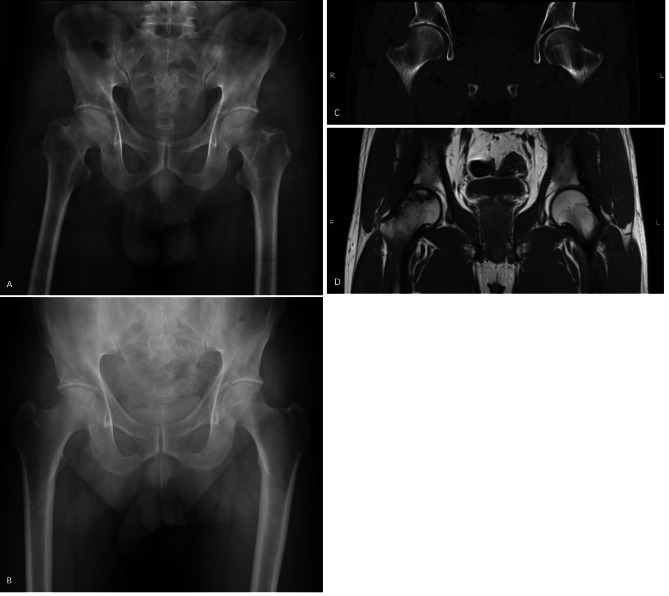
A 52-year-old male patient with ONFH of his right hip. He had undergone CD surgery 2 years previously. (A) Nevertheless, no evidence of a subchondral fracture, a fracture in the necrotic portion or flattening of the femoral head was observed. (B) Preoperative X-rays. (C) Preoperative CT. (D) Preoperative MRI.

The CD was considered to have failed if collapse of the femoral head was identified within two years after the initial surgery or if the patient underwent arthroplasty with artificial joint replacement during the follow-up period, even if there was femoral head integrity. Otherwise, the CD was considered to be successful regardless of the pain and functional status of the patient. Collapse was defined as the presence of femoral head depression > 2 mm according to radiographs (Figs. 2 and 3).

### Statistical analysis

Excel 2016 for Windows (Microsoft Corporation, Seattle, WA, USA) and SPSS 19.0 statistical software for Windows (IBM, Armonk, NY, USA) were used for statistical analysis, and univariate analysis was used to screen out the factors related to femoral head collapse after decompression surgery. Categorical variables are expressed as frequencies. Continuous variables are expressed as the mean ± SD. The Student’s t and chi-square test method were used. Subsequently, predictors with P < 0.05 were included in the Cox analysis. Cox analysis was used in the multivariate and multivariate survival analyses (P < 0.05 with significant difference). The influencing factors with statistical significance were screened out one by one. All filtered continuous variables were assessed by receiver operating characteristic (ROC) curves. The cut-off values were decided according to the assessment of the ROC curves. For each variable, three cut-off points were performed with an estimated risk of 25%,50% and 75% for CD failure from the coordinate points of the ROC curve. Ultimately, each continuous variable is divided into four levels of categorical variables. The multivariate Cox regression analysis compared the converted categorical variables between the patients with collapsed versus non-collapsed outcomes. The final model was used to predict femoral head collapse, and the sensitivity/specificity/ROC curves were produced. A P value < 0.05 was considered statistically significant. We developed a predictive scoring system using the results of the multivariate Cox regression model to show the hazard ratio and β of each predictor and to assess the probability of collapse. Furthermore, a weighted scoring system was generated to predict the hip collapse probabilities.

## Results

### Baseline characteristics of the patients

The study finally included 1537 hips after decompression surgery. The overall failure rate of the femoral head surgery was 52.44% at the two-year follow-up. None of the demographic and baseline characteristics differed between the 2 groups, including the body mass index, the affected side, hypertension, diabetes, corticosteroid treatment, osteoporosis, dialysis, vasculitis, arterial thrombosis, immune system diseases, preoperative treatment with hip preservation, preoperative crutch use, the ARCO stage, the VAS score, total cholesterol level, Harris hip score and number of channels. Significant differences were observed between the two groups in age, sex, aetiology, occupation, smoking, alcohol abuse, triglyceride level, disease duration and haemoglobin level. The patients’ baseline characteristics are summarized in Tables 1 and 2.

**Table 1:** Demographic features of the patients undergoing CD

Patient characteristics		Total (n = 1537)	Noncollapsed group (n = 731)	Collapsed group (n = 806)	Statistical value	P
Age (years)		43.11 ± 13.13	42 ± 13.60	44.12 ± 12.61	-3.160	0.002
Sex	Male	1211 (78.8%)	511 (69.9%)	700 (86.8%)	65.859	< 0.001
	Female	326 (21.2%)	220 (30.1%)	106 (13.2%)		
Body mass index (kg/m²)		25.10 ± 3.15	25.18 ± 3.22	25.03 ± 3.10	0.923	0.356
Aetiology	Alcohol-induced	693 (45.1%)	305 (41.7%)	388 (48.1%)	21.890	< 0.001
	Idiopathic	768 (50%)	404 (55.3%)	364 (45.2%)		
	Corticosteroid-induced	76 (4.9%)	22 (3%)	54 (6.7%)		
Side	Bilateral	1260 (82%)	594 (81.3%)	666 (82.6%)	0.488	0.485
	Unilateral	277 (18%)	137 (18.7%)	140 (17.4%)		
Occupation	Weight-bearing	591 (38.5%)	300 (41%)	291 (36.1%)	41.914	< 0.001
	Sitting	477 (31%)	170 (23.3%)	307 (38.1%)		
	Standing	469 (30.5%)	261 (35.7%)	208 (25.8%)		
Smoker	NO	811 (52.8%)	434 (59.4%)	377 (46.8%)	24.405	< 0.001
	YES	726 (47.2%)	297 (4.6%)	429 (53.2%)		
Alcohol use	NO	957 (62.3%)	508 (69.5%)	449 (55.7%)	31.010	< 0.001
	YES	580 (37.7%)	223 (30.5%)	357 (44.3%)		

^*^Mann–Whitney U test

^#^Chi-square test

**Table 2:** Potential risk factors for treatment failure in patients undergoing CD

Feature of the patients		Total (n = 1537)	Noncollapsed group (n = 731)	Collapsed group (n = 806)	Statistical value	P
Hypertension	NO	1376 (89.5%)	656 (89.7%)	720 (89.3%)	0.069	0.793
	YES	161 (10.5%)	75 (10.3%)	86 (10.7%)		
Diabetes	NO	1497 (97.4%)	718 (98.2%)	779 (96.7%)	3.735	0.053
	YES	40 (2.6%)	139 (1.8%)	27 (3.3%)		
Corticosteroid treatment	NO	1390 (90.4%)	664 (90.8%)	726 (90.1%)	0.256	0.613
	YES	147 (9.6%)	67 (9.2%)	80 (9.9%)		
Osteoporosis		0.19 ± 1.38	0.18 ± 1.37	0.19 ± 1.39	-0.054	0.957
Total cholesterol (mmol/L)		4.42 ± 1.04	4.44 ± 1.04	4.41 ± 1.04	0.540	0.589
Triglycerides (mmol/L)		1.51 ± 0.84	1.58 ± 0.89	1.45 ± 0.80	2.828	0.005
Haemoglobin (g/L)		139.12 ± 15.48	142.01 ± 15.92	136.50 ± 14.59	7.087	< 0.001
VAS score		4 ± 2.47	3.98 ± 2.50	4.02 ± 2.45	-0.321	0.749
Harris hip score		71.52 ± 11.55	72.93 ± 12.88	70.24 ± 11.11	4.596	0.721
Number of channels	2	524 (34.1%)	264 (36.1%)	260 (32.3%)	2.538	0.111
	3	1031 (67.1%)	467 (63.9%)	546 (67.7%)		
Disease duration (months)		4.37 ± 5.24	3.49 ± 4.33	5.17 ± 5.85	-6.425	< 0.001
Dialysis	NO	1527 (99.3%)	727 (99.5%)	800 (99.3%)	0.231	0.631
	YES	10 (0.7%)	4 (0.5%)	6 (0.7%)		
Vasculitis	NO	1532 (99.7%)	729 (99.7%)	803 (99.6%)	0.115	0.735
	YES	5 (0.3)	2 (0.3%)	3 (0.4%)		
Arterial thrombosis	NO	1532 (99.7%)	728 (99.6%)	804 (99.8%)	0.311	0.577
	YES	5 (0.3%)	3 (0.4%)	2 (0.2%)		
Immune system diseases	NO	1511 (98.3%)	722 (98.8%)	789 (97.9%)	1.777	0.183
	YES	26 (1.7%)	9 (1.2%)	17 (2.1%)		
Preoperative treatment with hip preservation	NO	997 (64.9%)	477 (65.3%)	520 (64.5%)	0.091	0.762
	YES	540 (35.1%)	254 (34.7%)	286 (35.5%)		
Preoperative crutch use	NO	1520 (98.9%)	723 (98.9%)	797 (98.9%)	0.002	0.967
	YES	17 (1.1%)	8 (1.1%)	9 (1.1%)		
ARCO stage	I	374 (24.3%)	180 (24.6%)	194 (24.1%)	0.064	0.800
	II	1163 (75.7%)	551 (75.4%)	612 (75.9%)		
Femoral head radius (mm)		25.99 ± 2.05	25.98 ± 2.10	26.00 ± 2.00	-0.230	0.818
Crescent sign	NO	1507 (98%)	722 (98.8%)	785 (97.4%)	3.783	0.052
	YES	30 (2%)	9 (1.2%)	21 (2.6%)		
Cystic changes of the femoral head	NO	879 (57.2%)	429 (58.7%)	450 (55.8%)	1.277	0.259
	YES	658 (42.8%)	302 (41.3%)	356 (44.2%)		
MRI feature-intensity	α	158 (10.3%)	93 (12.7%)	65 (8.1%)	16.195	< 0.001
	β	692 (45%)	345 (47.2%)	347 (43.1%)		
	γ	687 (44.7%)	293 (40.1%)	394 (48.9%)		
MRI feature-sum	I	751 (48.9%)	434 (59.4%)	317 (39.3%)	80.728	< 0.001
	II	190 (12.4%)	98 (13.4%)	92 (11.4%)		
	III	596 (38.8%)	199 (27.2%)	397 (49.3%)		
MRI feature-“double-line” sign	NO	315 (20.5%)	191 (26.1%)	124 (15.4%)	27.157	< 0.001
	YES	1222 (79.5%)	540 (73.9%)	682 (84.6%)		
MRI feature-bone marrow oedema	NO	971 (63.2%)	473 (64.7%)	498 (61.8%)	9.667	0.022
	I	53 (3.4%)	27 (3.7%)	26 (3.2%)		
	II	142 (9.2%)	50 (6.8%)	92 (11.4%)		
	III	371 (24.1%)	181 (24.8%)	190 (23.6%)		
MRI feature-articular effusion	NO	529 (34.4%)	267 (36.5%)	262 (32.5%)	2.743	0.098
	YES	1008 (65.6%)	464 (63.5%)	544 (67.5%)		
The combined necrosis angle		167.70 ± 83.37	131.59 ± 77.13	200.44 ± 74.91	-17.742	< 0.001
Articular glenoid labral tear	NO	1487 (96.7%)	705 (96.4%)	782 (97%)	0.408	0.523
	YES	50 (3.3%)	26 (3.6%)	24 (3%)		
Acetabular impingement syndrome	NO	777 (50.6%)	381 (52.1%)	396( 49.1%)	1.370	0.242
	YES	760 (49.4%)	350 (47.9%)	410 (50.9%)		

^*^Mann–Whitney U test

^#^Chi-square test

VAS, visual analogue scale; ARCO, Association Research Circulation Osseous; MRI, magnetic resonance imaging

In terms of the radiological measurements, none of the following radiological characteristics differed between the 2 groups: crescent sign, cystic changes of the femoral head, MRI feature-articular effusion, articular glenoid labral tear, acetabular impingement syndrome and femoral head radius. Significant differences were observed between two groups in the MRI features “double-line” sign, intensity, sum, and bone marrow oedema, as well as the combined necrosis angle. The patients’ radiological characteristics are summarized in Table 2.

### The failure rate of core decompression and risk factors

Among all 1537 hips, a failed CD surgery was identified in 806 hips. The overall failure rate of CD surgery was 52.44%.

Seven independent risk factors for failed CD surgery were identified via multivariate Cox regression. Male sex (HR = 75.449; 95% CI, 42.863-132.807) and Aetiology (Idiopathic HR = 2.762; 95% CI, 2.016–3.788, Steroid-induced HR = 2.543; 95% CI, 1.852–3.685) were risk factors for failed CD surgery. In terms of occupation, compared to the patients who stood at their occupation, the patients who were seated at their occupation were more likely to experience a failed CD surgery (HR = 3.937; 95% CI, 2.712–5.716). Age was another risk factor related to a failed CD surgery. With every yearly increase in age, the possibility of failed CD surgery increased by 4.5% (HR = 1.045; 95% CI, 1.032–1.058). The haemoglobin levels were another risk factor related to failed CD surgery. With every 1 g/L decrease in the haemoglobin levels, the possibility of failed CD surgery increased by 9.1% (HR = 0.909; 95% CI, 0.897–0.922). The duration of disease (HR = 1.217; 95% CI, 1.169–1.267) and the combined necrosis angle (HR = 1.025 degrees; 95% CI, 1.022–1.028) were risk factors for failed CD surgery. (Table 3)

**Table 3:** The multivariate Cox regression analysis and the receiver operating characteristic curve between two groups

Risk (protective) factor	Hazard ratio	95% CI for hazard ratio	β-coefficient	P	Area under curve
Sex					0.599(0.571–0.627)
Female					
Male	75.449	42.863-132.807	4.323	< 0.001	
Age (years)	1.045	1.032–1.058	0.044	< 0.001	0.554(0.525–0.582)
Occupation					0.484(0.455–0.514)
Weight-Bearing					
Standing	0.887	0.615–1.280	-0.120	0.523	
Seatting	3.937	2.712–5.716	1.370	< 0.001	
Haemoglobin (g/L)	0.909	0.897–0.922	-0.095	< 0.001	0.361(0.333–0.389)
Duration of disease (months)	1.217	1.169–1.267	0.196	< 0.001	0.642(0.614–0.669)
Aetiology					0.466(0.437–0.495)
Alcohol-induced (ref.)					
Idiopathic	2.762	2.016–3.788	1.017	< 0.001	
Steroid-induced	2.543	1.852–3.685	0.853	< 0.001	
The combined necrosis angle	1.025	1.022–1.028	0.024	< 0.001	0.775(0.751–0.798)

CI, confidential interval

### Predictive model and scoring system

After the determination of the cut-off points of continuous variables by using ROC curves, a predictive mode was set up to help build the scoring system. The new multivariate Cox regression analysis compared the converted categorical variables between the patients with collapsed and non-collapsed outcomes (Table 4).

**Table 4:** Results of the multivariate Cox regression analysis comparing the converted categorical variables between two groups

Risk (protective) factor	Hazard ratio	95% CI for hazard ratio	β-coefficient	P
Sex				
Female				
Male	297.490	137.133–645.360	5.695	< 0.001
Age (years)				
<30				
<39	3.000	1.744–5.159	1.099	< 0.001
<53	7.637	4.425–13.179	2.033	< 0.001
≥53	6.974	3.831–12.697	1.942	< 0.001
Occupation				
Weight-Bearing				
Standing	1.155	0.748–1.784	0.144	0.515
Seatting	7.617	4.754–12.206	2.030	< 0.001
Haemoglobin (g/L)				
≥154.2				
<154.2	3.314	1.873–5.862	1.198	< 0.001
<145.5	13.304	7.333–24.137	2.588	< 0.001
<134	373.617	163.716-852.629	5.923	< 0.001
Duration of disease (months)				
<1				
<2.1	0.518	0.298–0.898	-0.658	0.019
<3.9	0.835	0.470–1.486	-0.180	0.541
≥3.9	26.657	14.327–49.599	3.283	< 0.001
Aetiology				
Alcohol-induced				
Idiopathic	5.525	2.257–13.514	1.710	< 0.001
Steroid-induced	4.852	1.587–9.265	1.352	0.478
The combined necrosis angle				
<72°				
<117°	3.203	1.475–6.953	1.164	0.003
<168°	33.120	15.903–68.974	3.500	< 0.001
≥168°	1154.011	466.923-2852.166	7.051	< 0.001

CI, confidential interval

**Fig. 4:**
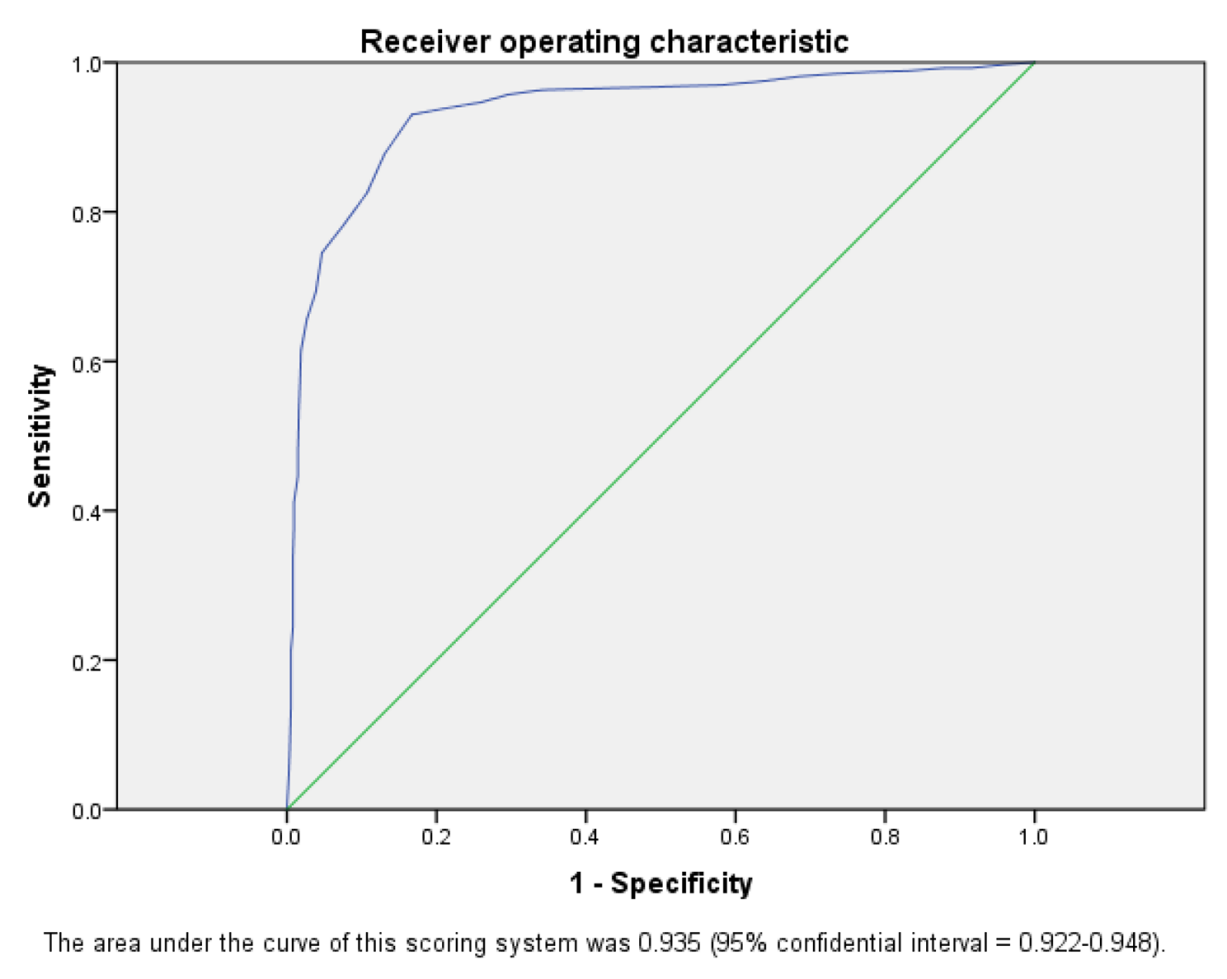
The area under the curve of this scoring system was 0.935 (95% confidential interval = 0.922–0.948) according to the assessment of the ROC curves

Subsequently, a weighted scoring system (Table 5) was generated according to the β-coefficients in the above Cox regression model. Seven items were included in this scoring system, such as sex (male = 6 points, female = 0 points), age (< 30 years = 0 points, < 39 years = 1 point, < 53 years = 2 points, ≥ 53 years = 2 points), occupation (weight-bearing = 0 points, seated = 2 points, standing = 0 points), haemoglobin level (≥ 154.2 g/L = 0 points, < 154.2 g/L = 1 point, < 145.5 g/L = 2.5 points, < 134 g/L = 6 points), duration of disease aetiology (< 1 month = 0 points, < 2.1 months = 0 points, < 3.9 months = 0 points, ≥ 3.9 months = 3 points), aetiology (idiopathic = 2 points, steroid-induced = 2 points, alcohol-induced = 0 points) and MRI necrosis angle (< 72° = 0 points, < 117° = 1 point, < 168° = 3.5 points, ≥ 168° = 7 points). The area under the curve of this scoring system was 0.935 (95% confidential interval = 0.922–0.948) according to the assessment of the ROC curves (Fig. 4). The predictive scores for a low risk (an estimated possibility of treatment failure ≤ 30%), moderate risk (an estimated possibility of treatment failure 31 − 69%), and high risk (an estimated possibility of treatment failure ≥ 70%) for the possibility of a treatment failure were ≤ 9 points, 10–15 points and ≥ 16 points, respectively. Furthermore, each estimated risk of CD failure is shown in Table 6.

**Table 5:** Weighted scoring system for the outcome prediction of core decompression

Factor	Score
Sex	
Female	0
Male	6
Age (years)	
<30	0
<39	1
<53	2
≥53	2
Occupation	
Weight-Bearing	0
Seatting	2
Standing	0
Haemoglobin (g/L)	
≥154.2	0
<154.2	1
<145.5	2.5
<134	6
Duration of disease (months)	
<1	0
<2.1	0
<3.9	0
≥3.9	3
Aetiology	
Alcohol-induced	0
Idiopathic	2
Steroid-induced	2
The combined necrosis angle	
<72°	0
<117°	1
<168°	3.5
≥168°	7

**Table 6:** Relationship between the estimated risk of treatment failure and the scores of the patients

Score	Estimated risk of treatment failure
7.5	< 10
10	< 20
11	< 30
12.5	< 40
14	< 50
15	< 60
17	< 70
18.5	< 80
20.5	< 90
> 20.5	100

## Discussion

In this study, the overall survival rate of the femoral head was 47.56% at the two-year follow-up, which was a moderate level compared to some similar studies. For instance, Yoon et al. [12] reported that the prevalence of failed CD surgery was 56.41%, and D’Ambrosi et al. [6] reported that the prevalence was up to 50%. Thus, approximately half of the CD surgeries had failed, and the patients had to undergo a second-stage hip arthroplasty. Incorporating cell-based components such as bone marrow stem cells, platelet-rich plasma or tantalum rods into the tract created by drilling is performed adjuvant to CD with varying success rates. For instance, Kang et al. [20] reported that the prevalence of failed CD combined with stem cells was 20%. In 10 studies published since 1996, 530 successful clinical results of 782 hips undergoing multiple CD with small diameter Steinmann pins were reported, with an overall failure rate of 32% [21]. Trousdale et al. observed a 58% failure rate postoperatively among patients in the CD group compared to a significantly lower value of 20% in the free vascularized fibular graft group [22]. Although CD in combination with bone graft, bone marrow injection, platelet-rich plasma injection or mesenchymal stem cell injection have achieved excellent clinical outcomes in the treatment of ONFH, some reports note complication rates as high as 10–15% [23]. Moreover, the identification of which patients would benefit from CD surgery is crucial for proper clinical decisions to be made that will improve the outcomes of patients. Based on the guidance of the Transparent Reporting of a multivariable prediction model for Individual Prognosis Or Diagnosis(TRIPOD) statement, a clinical protocol was established to predict the potential failure of CD surgery in patients with ARCO stage I-II ONFH in the present study.

Prior to the application of this scoring system, an understanding of the independent risk factors for CD failure by surgeons is important. In this study, seven risk factors were determined: male sex, age, an occupation that was mainly performed while sitting, a lower haemoglobin concentration, a long-term duration of the disease, steroid-induced osteonecrosis and an increased combined necrosis angle. Among these risk factors, male sex, an older age and the necrosis angle have already been well established [24–27]. For example, Serong et al. reported that the reduced ability of bone marrow stromal cells to differentiate into osteoblasts in elderly patients significantly diminished the healing capacity of bone, resulting in an insufficient regeneration of the femoral head after necrosis [7]. In another study from Boontanapibul K et al. [28], the addition of BMAC had more reliable outcomes than isolated core decompression for precollapse ONFH if the combined necrotic angles were < 250°. A large volume of necrosis on MRI and a high combined necrotic angle were risk factors for failed CD surgery [29–31]. However, some risk factors have not been previously studied in depth, including the seating status of the patient’s occupation, a lower haemoglobin concentration and steroid-induced osteonecrosis. In contrast to our study, Bozic KJ et al. [32] reported that patients who have an acute onset of symptoms may have a more rapidly progressive form of the disease, and this may be a predictor of adverse outcomes after core decompression. Although other authors have suggested that outcomes are worse for patients who have steroid-related nontraumatic osteonecrosis [33–35], no previous study, to our knowledge, has demonstrated an independent statistical relationship between the use of steroids and the survival of the hip. In previous studies, very few investigators have explored the relationship between the sitting position and CD surgical outcomes. Researchers postulated that longer seated periods are associated with clinical fracture [36]. Anatomically and biomechanically, load of the femoral head increases during femoral head injury in a sitting position due to the backward forces of the femur [37]. Our study shows that the sitting position is a risk factor. However, this specific mechanism still requires further verification by conducting numerous studies.

Note that the key word “independent” is emphasized when these risk factors were discussed because many reports have demonstrated that the prognosis after CD is affected by different factors. For instance, Mont et al. reported that the location of the necrotic lesions relative to the acetabular weight-bearing portion is associated with failure of CD surgery [38], Classen T et al. reported that the lesion size is a predictive factor for CD failure in patients [39], and Serong et al. reported that femoroacetabular impingement syndrome is associated with the failure of CD surgery [40]. However, instead of performing a comprehensive evaluation that includes several factors, the common shortcomings of these previous studies were that only one or two factors were included in each study. Therefore, the prognosis of patients after CD could not be fully predicted. In this study, all seven independent risk factors (both clinical factors and radiological factors were all included) were weighted and included in the same predictive model. In addition, the colinear biases were eliminated by stepwise regression. Consequently, this scoring system could comprehensively predict the prognosis of patients undergoing CD. This is the most important novelty of this study. The results showed an excellent-good predictability of this scoring system (area under curve = 0.935). Meanwhile, for each estimated risk, the corresponding cut-off point was given (Table 6). Therefore, clinical surgeons could use this protocol to estimate the risk of CD failure in a certain patient. We believe this will provide great help in determining whether a patient should receive a CD or a one-stage hip arthroplasty. We recommend that for patients with ARCO stage I - II ONFH, this protocol should be used before the final clinical decision is made.

There are several limitations of this study. First, as a single-centre study with all surgeries performed by the same group of surgeons, the effect of the surgical skills of the surgeons could not be evaluated. Second, the patients were asked to recall their history of ONFH, which could have caused a recall bias. Some potentially meaningful indicators, such as the total alcohol consumption, total smoking, and the total hormone treatments, are not available in a clinically accurate manner. Third, the scoring system was established and evaluated using the same group of patients, which made the evaluation results less accurate, and a prospective research cohort needs to be established to further validate the model’s accuracy. Fourth, in this study if a patient received bilateral CDs, he or she was considered two independent individuals. This might finally result in some statistics error, since the demographic characteristics of these patients were doubled.

## Conclusions

Seven independent prognostic factors were identified in this study: male sex, an older age, the seating status of the patient’s occupation, a lower haemoglobin concentration, a long-term disease duration, aetiology and an increased combined necrosis angle. By comprehensively including and weighing these risk factors, a new scoring system was built, which showed a good predictivity for a core decompression failure. This new scoring system might provide evidence-based medical proof for determining whether a patient with ARCO stage I - II ONFH might benefit from CD surgery. This scoring system is crucial for making clinical decisions.

## Data Availability

and materials. All the data generated or analysed during this study are included in this published article.
